# Role of inhibin and activin in the modulation of gonadotropin- and steroid-induced oocyte maturation in the teleost Fundulus heteroclitus

**DOI:** 10.1186/1477-7827-5-21

**Published:** 2007-06-05

**Authors:** Teresa R Petrino, Gesulla Toussaint, Yu-Wai P Lin

**Affiliations:** 1 Barry University, School of Natural & Health Sciences, Miami Shores, Florida 33161, USA

## Abstract

**Background:**

Activin and inhibin are glycoproteins structurally related to the transforming growth factor-beta superfamily. These peptides were first described as factors that regulate the follicle-stimulating hormone (FSH) at the pituitary level. The possible role of inhibin and activin, at the ovarian level, in mediating the stimulatory actions of a Fundulus pituitary extract (FPE) and 17alpha,20beta-dihydroprogesterone (DHP) on oocyte maturation was investigated in this study.

**Methods:**

In vitro culture of ovarian follicles and induction of oocyte maturation were carried out in 75% Leibovitz L-15 medium. Follicles or denuded oocytes were exposed to FPE, inhibin, activin, ethanol vehicle (control group), or DHP. The competence of the follicles or denuded oocytes to respond to the hormones was assessed by scoring germinal vesicle breakdown (GVBD) used as an indication of the reinitiation of meiosis or oocyte maturation. DHP level was measured by radioimmunoassay.

**Results:**

Addition of FPE promoted the synthesis of DHP by the granulose cells of fully grown ovarian follicles and thus stimulated GVBD in the oocyte. Presence of porcine inhibin did not hinder the synthesis of DHP stimulated by FPE, although it did inhibit the subsequent GVBD in a dose-dependent manner, suggesting that the action of inhibin was at the oocyte level. Similarly to the findings with FPE, inhibin also blocked the DHP-induced GVBD in intact follicles, as well as the spontaneous and steroid-induced GVBD of denuded oocyte. Inhibin straightforwardly blocked the response to a low dose of DHP throughout the culture period, while higher doses of the steroid appeared to overcome the inhibitory effect especially at later times. In contrast to inhibin, recombinant human activin A significantly enhanced DHP-induced GVBD in a dose-dependent manner after 48 hr, although activin alone was not able to induce GVBD without the presence of the steroid.

**Conclusion:**

Taking together with our previous studies that demonstrate the presence of activin/inhibin subunits in the ovary of F. heteroclitus, these in vitro findings indicate that inhibin and activin are local regulators in the teleost ovary and have opposing effects in modulating oocyte maturation.

## Background

Activin and inhibin are peptides structurally related to the transforming growth factor-β (TGF-β) superfamily of proteins. Inhibins are heterodimeric glycoproteins composed of an α-subunit and one of several forms of β-subunits (e.g. βA or βB), resulting in biologically active forms termed inhibin A and inhibin B. Activins are composed of two β subunits in any combination [1]. These peptides, found in mammalian follicular fluids, were first described as factors that regulate the follicle-stimulating hormone (FSH) at the pituitary level [2-4]. Cumulative evidence further has established that activin and inhibin function also as local autocrine/paracrine regulators in the gonads. Indeed, these peptides have been implicated in an array of processes in the ovary of mammals including follicle recruitment, granulosa and theca cell proliferation and atresia, steroidogenesis, ovulation, and luteinization [5-7]. In addition, activin and inhibin have been implicated in oocyte maturation, albeit conflicting evidence has been reported. In this regard, inhibin was shown to have no effect [8] or to inhibit spontaneous division in both cumulus enclosed and denuded oocytes from immature rats [9] and to suppress luteinizing hormone-induced meiosis in follicle-enclosed oocytes of preovulatory rats [8]. Activin was reported to have no effect on oocyte maturation in rats [9] and pig [10], but it has been shown to increase oocyte maturation in immature rats [11,12], monkeys [13], cows [14] and humans [15]. Compared to the information available for mammalian species, less is known about the effect of TGF-β related peptides in lower vertebrates. Yet, lower vertebrates offer large numbers of ovarian follicles and therefore they have already served as excellent models for the study of oocyte development and maturation. Indeed, it is well known that in lower vertebrates, oocyte maturation is triggered by a surge of gonadotropin hormones that, acting in the granulose cells, increases the production of progestogens, which action on the oocyte initiates germinal vesicle breakdown (GVBD), or oocyte maturation.

In fish, all major components of the activin-inhibin-follistatin system have been identified [16]. In fact, several studies have shown that inhibin/activin β-subunits are expressed in goldfish [17-19]; rainbow trout [20]; mullet [21]; zebrafish [22-25]; and killifish [GenBank:AF503775, GenBank:DQ149108, GenBank:DQ387061]. So far, there is evidence for two isoforms of activin βB and two isoforms of βA in teleost. Furthermore, recombinant goldfish activin A (βA βA) and activin B (βB βB) with biological activities were prepared and used for physiological studies [26]. However, while the biological activity of activin has been documented particularly in goldfish at the level of the pituitary regulation of gonadotropin [27], and in zebrafish at the ovarian level [25,28-30], the biological activity of inhibin was not investigated in fish and it is much less understood. Only recently, the complete inhibin α-subunit has been cloned or deduced for three species of fish *Oncorhynchus mykiss *(rainbow trout) [GenBank:AB044566], *Fundulus heteroclitus *(killifish) [GenBank:AY836522], and *Danio rerio *(zebrafish) [GenBank:NM_001045204], thus indicating that different assembled dimeric inhibin/activin isoforms may be present in the fish ovary. The aim of this study was to explore and characterize the effects of inhibin on both steroidogenesis and oocyte maturation, or GVBD, in *F. heteroclitus*. In addition, activin was also used in this study, and results show that while activin alone does not initiate GVBD, inhibin and activin have mutually opposing actions to modulate steroid-induced oocyte maturation.

## Methods

### Animal

Killifish (*Fundulus heteroclitus*) were collected from salt marshes in the vicinity of St. Augustine, Florida. In the laboratory, fish were maintained in a 25 gal. aquarium at 25°C on a 14/10 hr light/dark cycle, and were fed three times a day with flake food (TetraMin). The care and use of, as well as all procedures involving, animals have been approved by Barry University's Institutional Animal Care and Use Committee (IACUC), in accordance with the guidelines of the IACUC of the National Institutes of Health (NIH). Fish were anesthetized in 100 ppm 3-aminobenzoic acid ethyl ester methanesulfonate salt (MS-222; Sigma, St Louis, MO) before being killed.

### Hormones and test substances

The effect of gonadotropin was tested by using a *F. heteroclitus *pituitary extract (FPE) prepared as described by [31] at a concentration of 10 pituitary equivalents/ml and kept in 100-μl aliquots at -20°C. Porcine inhibin (Sigma, St. Louis, MO) [with no specification of what form of inhibin (inhibin A or inhibin B or a mixture of both) is present in the preparation], recombinant human activin A [#15365-36(1)], and recombinant human inhibin A [#NU1-4315] that were supplied by Dr. A. F. Parlow (NIDDK's National Hormone and Pituitary Program and NICHD), were dissolved in culture media, aliquoted, and kept at -20°C. FPE, inhibin, and activin aliquots were thawed only once before each experiment and added directly to the culture to obtain the desired concentration. The steroid 17α,20β-dihydroprogesterone (DHP) was obtained from Steraloids Inc. (Newport, RI), dissolved in ethanol, and added (5 μl) directly to the culture medium.

### In vitro culture of ovarian follicles and induction of oocyte maturation

Ovaries were removed from females and placed in 75% Leibovitz L-15 medium with L-glutamine (Sigma) containing 100 μg gentamicin/ml, and adjusted to pH 7.5 with HCl [32]. Intact fully grown prematurational follicles (1.2–1.4 mm in diameter), with visible germinal vesicle, were manually isolated from several ovaries with the aid of fine forceps under a stereomicroscope. *F. heteroclitus *intact follicles are surrounded by a single layer of granulosa cells external to the vitelline envelope, a vascularized connective tissue sheath or theca, and a simple surface epithelium [33]. Denuded oocytes (without the enveloping follicular cell layers) were obtained by a combination of manual dissection to remove the epithelium and theca layers, and treatment with Ca^2+^/Mg^2+^-free medium to remove the granulosa cells according to [34]. In both cases, intact follicles or denuded oocytes (pooled from 2–4 ovaries) were washed several times with fresh medium during the isolation procedure. After 1 hr at room temperature (22–25°C), the atretic oocytes were discarded, and the remaining healthy follicles or oocytes were randomly distributed into 24-well tissue culture trays (Costar No.3525). Each culture well contained 20 intact follicles/1 ml L-15 media or 10–14 denuded oocytes depending of the experiment. Follicles or denuded oocytes were then exposed at time zero to FPE, inhibin, activin, ethanol vehicle (control group), or DHP, identified as the maturational inducing substance (MIS) in *F. heteroclitus *[35]. Incubations were carried out at room temperature for up to 72 hr with no subsequent hormone addition or media change as previously described [31]. The competence of the follicles or denuded oocytes to respond to the hormones was assessed by scoring germinal vesicle breakdown (GVBD) used as an indication of the reinitiation of meiosis or oocyte maturation [36] several times up to 72 hr.

### DHP Radioimmunoassay (RIA)

Previous data for this species [31] have shown that after FPE stimulation the maximum level of steroid production occurs around 24 hr and it is prior to the occurrence of GVBD, which maximum response takes place around 72 hr. Based on these findings, aliquots of culture medium were removed at 24 hr and directly assayed for DHP as previously described [37]; while GVBD was monitored up to 72 hr of culture in the same group of follicles. Radiochemical used as tracer for the DHP radioimmunoassay was obtained from New England Nuclear, Boston, MA, and prepared as described by [31]. Antiserum against DHP was a gift from Dr. Y. Nagahama (Japan).

### Statistics

Data are presented as mean ± SEM from three or more experiments performed at different dates. Statistical comparisons were conducted by analysis of variance, and the means were subsequently compared by Tukey's test or Hall-Sidak method (all pairwise multiple comparisons). Differences were considered significant if P ≤ 0.05.

## Results

### Inhibin effects on oocyte maturation induced by gonadotropin

**Figure 1:**
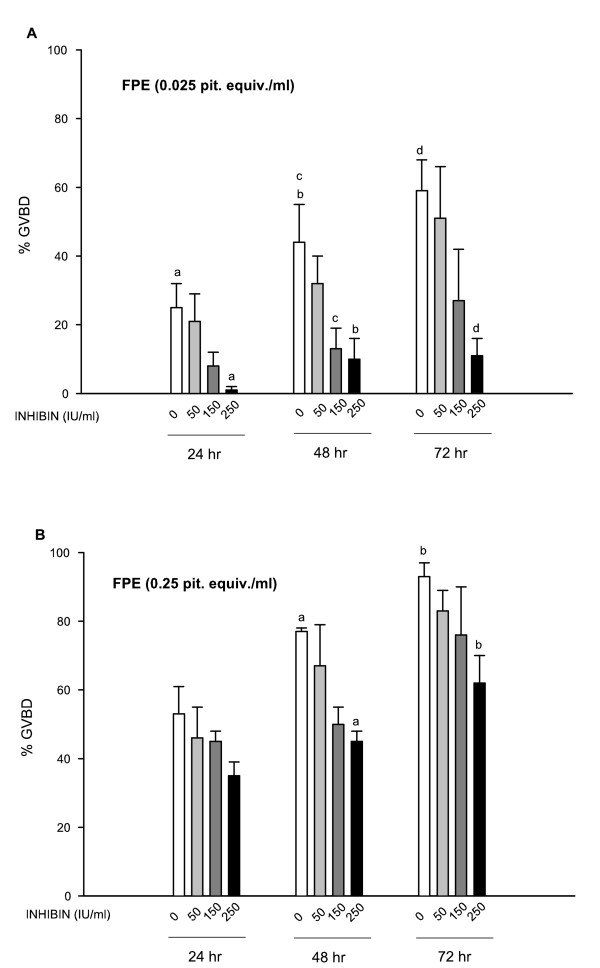
Effects of inhibin on FPE-induced oocyte maturation. Isolated ovarian follicles were treated with two doses of FPE: 0.025 pit. equiv./ml (**A**) or 0.25 pit. equiv./ml (**B**) alone or in the presence of inhibin (50–250 IU/ml) as indicated on the abscissa. Inhibin was added simultaneously or 1 hr previous to FPE addition. Oocyte maturation was monitored by scoring GVBD at 24, 48, and 72 hr of incubation, also indicated on the abscissa. Data are the mean ± SEM from four different experiments. Same letter indicates significantly different from each other at *P *≤ 0.05.

It has been well documented that addition of FPE (used as a source of homologous gonadotropin hormones) to *F. heteroclitus *intact follicles cultured in vitro promotes an increase in the synthesis of 17α,20β-dihydroprogesterone (DHP) by the granulosa cells [33,38]. This steroid, DHP, has been demonstrated to be the natural maturation inducing substance in this species [35], and it acts directly on the oocyte to induce GVBD. To investigate whether the gonadal peptide inhibin can affect these follicular responses, follicles were incubated with various doses of FPE and purified porcine inhibin. Results (Fig. 1) show that inhibin significantly reduced the number of follicles that underwent GVBD in response to the low dose of FPE (0.025 pit. equiv./ml). The maximum response to FPE in terms of GVBD occurred at 72 hr, and a consistent dose-dependent inhibitory effect of inhibin was observed throughout the entire incubation period (Fig. 1A). On the other hand, the response of the follicles to a higher dose of FPE (0.25 pit. equiv./ml) was affected to a lesser extent by inhibin (Fig. 1B). Only the higher dose of inhibin (250 IU) caused a significant but not complete inhibition in the GVBD response (Fig. 1B).

**Figure 2:**
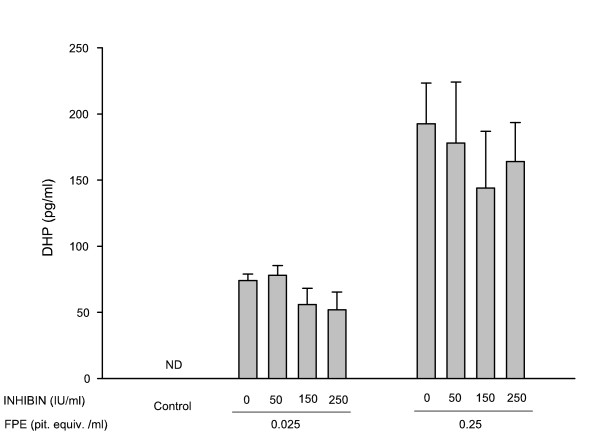
Effects of inhibin on FPE-induced steroid secretion. After 24 hr of culture with the various treatments described in Fig. 1, a fraction of the media was collected for determination of steroid level by radioimmunoassay. Values are the mean ± SEM from four different experiments. Control = follicles cultured in medium alone without FPE or inhibin. ND = not detectable.

In addition, radioimmunoassay of the culture media collected after 24 hr of incubations (Fig. 2), shows an FPE dose-dependent increase in the levels of DHP as previously demonstrated for this species [31]. Addition of inhibin (50–250 IU/ml) in combination with FPE (0.025 and 0.25 pit. equiv./ml) did not significantly affect the steroid levels induced by FPE stimulation. Addition of inhibin simultaneously or one hr previous to the addition of FPE has shown similar results (data not shown).

### Inhibin effects on oocyte maturation induced by steroid

**Figure 3:**
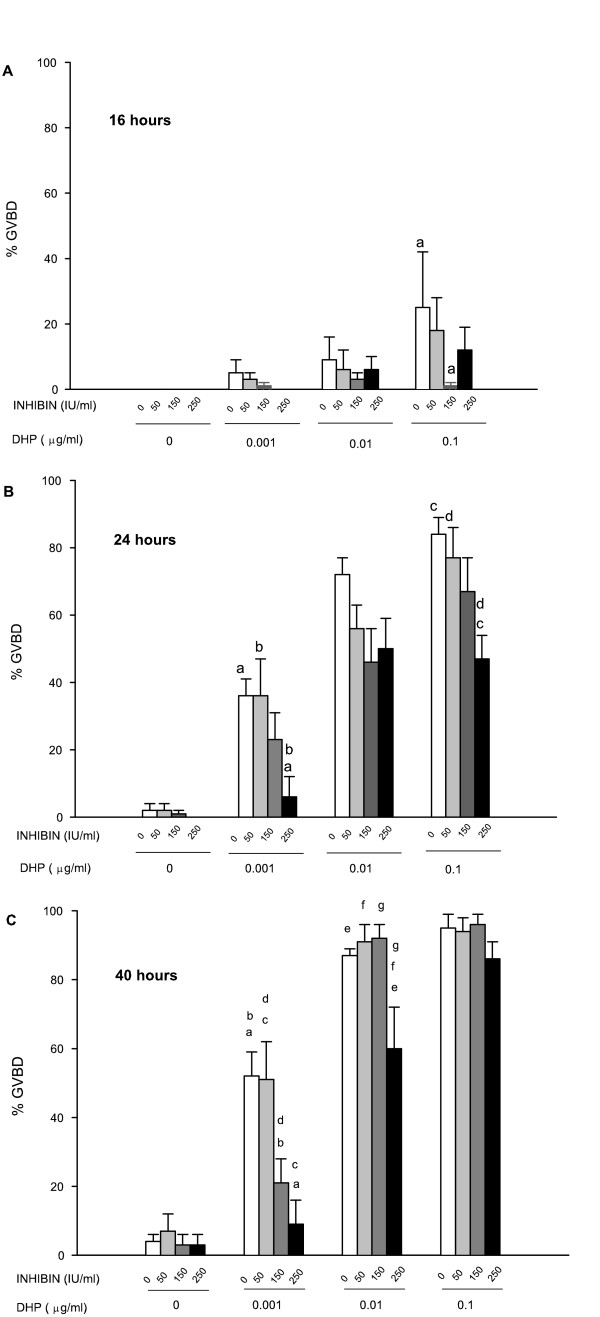
Effects of inhibin on DHP-induced oocyte maturation. Various doses of inhibin, as indicated on the abscissa, were added to the culture media together with three doses of DHP (0.001, 0.01, and 0.1) μg/ml. Oocyte maturation was monitored by scoring GVBD at 16 (**A**), 24 (**B**) and 40 hr (**C**) of incubation. Data are the mean ± SEM from 3–4 different experiments. Same letter indicates significantly different from each other at *P *≤ 0.05.

Because FPE induction of GVBD is mediated through the synthesis of DHP, and since the production of this steroid was not affected by inhibin (Fig. 2), the previous experiments suggest that inhibin may affect the process leading to GVBD initiated by the steroid on the oocyte. The next experiments were conducted to investigate the effect of inhibin on the GVBD response to exogenously added DHP. Results (Fig. 3) show that inhibin decreased DHP-induced GVBD during the first 24 hr of culture (Fig. 3A and 3B) at all concentration of DHP used. At this point in time, an apparent and significant dose-dependent inhibition caused by inhibin was observed when it was combined with the lowest dose (0.001 μg/ml) of DHP, while only a partial inhibition (approximately 30% less GVBD) was observed when inhibin was combined with higher doses of DHP (0.01 and 0.1 μg/ml) (Fig. 3B). Interestingly, after 40 hr of culture (Fig. 3C), when the maximum GVBD was achieved in response to the various doses of DHP alone, the inhibitory effect caused by inhibin, which was observed at earlier times, became less evident. No inhibition was observed with any of the inhibin doses (50–250 IU/ml) and the highest doses of DHP (0.1 μg/ml). Only the highest dose of inhibin (250 IU/ml) partially blocked the response (80% vs. 50% GVBD) to the middle dose of DHP (0.01 μg/ml). However, the inhibin dose-dependent inhibition only persisted with the lowest doses of DHP (0.01 μg/ml) throughout the entire culture. In contrast to the inhibitory action on the DHP-induced GVBD observed in the presence of porcine inhibin, we found that addition of recombinant human inhibin A had no effect on GVBD. It did not induce GVBD on its own; neither blocked nor enhanced DHP-induced GVBD (data not shown).

### Inhibin effects on denuded oocytes

**Figure 4:**
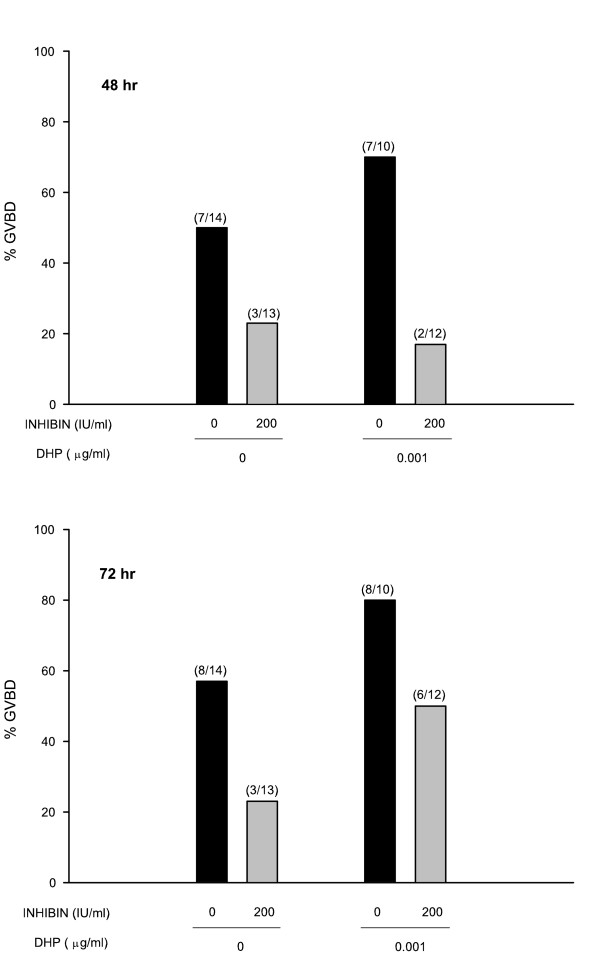
Effects of inhibin on denuded oocytes. Oocytes without cellular investments were treated with or without DHP (0.001 μg/ml) and/or inhibin (200 IU). In parentheses is the number of oocyte with GVBD/total number of oocytes per treatment.

To investigate whether the inhibition on the DHP-induced GVBD was the result of a direct interaction of inhibin with the oocyte or whether the inhibitory action was mediated through the follicular cells surrounding the oocyte other than the synthesis of DHP, we treated denuded oocytes (without cellular investments) with inhibin and DHP. In contrast to intact follicles, a higher incidence of GVBD (spontaneous maturation) is observed in *F. heteroclitus *denuded oocytes without exogenous stimulation [34]. Figure 4 show that inhibin blocked the spontaneous as well as the DHP-induced GVBD of denuded oocytes at both 48 hr and 72 hr of culture. However, the inhibitory effect was less pronounced at the latest time in the group of denuded oocytes induced by a low dose of DHP.

### Activin effects on oocyte maturation induced by steroid

**Figure 5:**
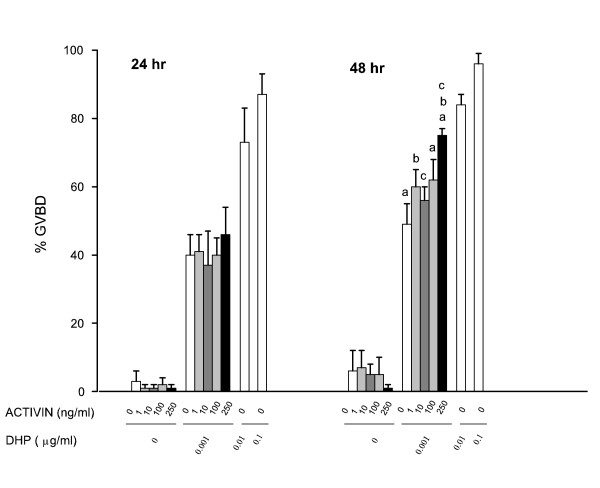
Effects of activin on DHP-induced steroid secretion. Various doses of human activin A (0–250 ng/ml), as indicated on the abscissa, were added to the culture media alone or in combination with a sub maximal dose of DHP (0.001 μg/ml). Follicles were also treated with higher doses of DHP (0.01 and 0.1 μg/ml) alone as positive controls. Oocyte maturation was monitored by scoring GVBD at 24 and 48 hr of incubation. Data are the mean ± SEM from 5 different experiments. Same letter indicates significantly different from each other at *P *≤ 0.05.

In contrast to inhibin, preliminary experiments indicated that activin may increase the number of oocytes undergoing GVBD. Thus, in order to explore the effect of activin on the DHP-induced GVBD, a sub maximal dose (0.001 μg/ml) of the steroid was used in combination with various doses of the gonadal peptide. Follicles from the same batch were also incubated with higher doses of DHP alone to get the maximal response. Results presented in Fig. 5 show that recombinant human activin A alone did not induce oocyte maturation (GVBD). Moreover, DHP-induced GVBD was not apparently affected by activin during the first 24 hr of culture (Fig. 5). However, activin A significantly enhanced, in a dose-dependent manner, the GVBD induced by a sub maximal dose of steroid (0.001 μg/ml) after 48 hr of culture. The highest dose of activin used (250 ng/ml) raised the GVBD values to a level similar to those induced by a dose of DHP ten times higher (0.01 μg/ml).

## Discussion

Results from the present study demonstrate that inhibin and activin have mutually opposing effects on modulating the process of oocyte maturation or GVBD induced by steroid in *F. heteroclitus*, thus implying a local role for these gonadal peptides in the fish ovary. This is supported by the findings that α- and β-subunits, which are the components of inhibin (dimmer of α and β subunits) and activin (dimmer of ββ subunits), are expressed in the ovary of this species [GenBank:AF503775, GenBank:DQ149108, GenBank:AY836522, GenBank:DQ387061].

Similar to other teleost, the process of GVBD in *F. heteroclitus *is initiated in the prophase I-arrested fully grown oocyte by gonadotropin stimulation of follicular DHP secretion [35]; this steroid (MIS) then acts on the oocyte to trigger the events leading to the reinitiation of meiosis. In the present study, data (Fig. 1) show that inhibin reduced FPE (used as a source of gonadotropin)-induced GVBD. The inhibitory effect on GVBD observed in the presence of inhibin was not only dependent on the inhibin dose but also on the FPE dose used to stimulate oocyte maturation. In effect, inhibin can readily block the stimulation of a low dose of FPE (0.025 pit. equiv./ml) but only moderately affects the response of the follicles to a higher dose of FPE (0.25 pit. equiv./ml) stimulation, suggesting that the inhibitory effect can be overcome by the action of gonadotropin. Furthermore, the action of inhibin on the FPE stimulation of GVBD is not apparently caused by a decrease in the production of the MIS by the granulosa cells. Data from the radioimmunoassay (Fig. 2) show that the DHP increase that follows FPE stimulation was not altered in the presence of porcine inhibin. It is important to note that medium steroid levels and GVBD were monitored simultaneously in the same set of follicles for each experiment. Since the action of FPE to induce GVBD is mediated through the synthesis of DHP, which was not affected by inhibin, it appears that the inhibin action to suppress GVBD in *F. heteroclitus *is more likely to occur at the oocyte level rather than to block steroid production by the follicle cells. In addition, we have previously shown that FPE initiates steroidogenesis in the *F. heteroclitus *ovarian follicles by mobilizing endogenous cholesterol into the mitochondria where the set of enzymes required for the synthesis of DHP are present even in unstimulated follicles [37]. Thus, results from this study indicate that inhibin does not affect the early steroidogenic pathway leading to DHP, the maturational steroid (MIS) in this species. Concomitant to DHP, *F. heteroclitus *follicles also synthesize testosterone and estradiol [31], which were not measured in this study, and thus we cannot discard the possibility that inhibin may affect other steroidogenic reactions. Interestingly, in a previous study from our laboratory [39] using intact follicles of the amphibian *Rana pipiens *(leopard frog), we have observed that porcine inhibin significantly blocks both the progesterone synthesis that follows gonadotropin stimulation and the subsequent GVBD. Similar to these findings in amphibians, inhibin has been shown to modulate steroidogenesis in avian species [40], and in mammalian species (for review see [6,41,42]. More data are necessary in other species of fish to investigate whether activin and inhibin can also serve as modulators of steroidogenesis in teleost.

Results from Fig. 3 show the dose-dependent inhibition of exogenously added DHP-induced GVBD by inhibin. Similar to the observations made for FPE, the inhibitory effect of inhibin on GVBD was very dependent not only on the inhibin dose but also on the doses of DHP used to trigger the maturational events. Inhibin straightforwardly blocked the response to a low dose of DHP (0.001 μg) throughout the culture period, while higher doses of the steroid appeared to overcome the inhibitory effect, especially at later times. The direct action of inhibin on the oocyte was also investigated in denuded oocytes (stripped of all follicular cells). It has been reported that removal of the granulosa cell layer from the oocyte of *F. heteroclitus *frequently triggers the resumption of meiosis in the absence of exogenous hormonal stimulation (spontaneous maturation) and thus it was postulated that granulosa cells are somehow responsible for the maintenance of meiotic arrest in non-hormone-treated oocytes [34]. As seen from the results depicted in Fig. 4, fifty percent of denuded oocytes underwent spontaneous maturation and they also responded to exogenously added DHP for the induction of GVBD. Addition of inhibin to denuded oocytes reduced both the spontaneous maturation as well as the maturation induced by DHP, indicating that inhibin acts directly on the oocyte to suppress GVBD. These results support the hypothesis postulated for other species that inhibin can act as a negative modulator of oocyte maturation. Indeed, bovine inhibin was reported to inhibit spontaneous oocyte maturation in both cumulus enclosed and denuded rat oocytes [9]; ovine inhibin and transforming growth factor beta (TGFβ) partially inhibited luteinizing hormone (LH)-induced meiosis in rat follicle-enclosed oocytes [8]; and porcine inhibin inhibited both the gonadotropin-stimulated progesterone production and the subsequent oocyte maturation in the amphibian *Rana pipiens *[39]. However, Wu *et al. *[30] have reported that recombinant human inhibin A stimulates GVBD in zebrafish follicles. Similarly to the zebrafish study, maturation-enhancing effects of recombinant human inhibin A were reported for primates [13]. We have also tried incubations of *F. heteroclitus *ovarian follicles with recombinant human inhibin A and found that it did not induce GVBD on its own; neither has it had an enhancement nor inhibitory effect on DHP-induced oocyte maturation (data not shown). Kagawa *et al. *[43] also reported that recombinant human inhibin A has no effect on oocyte maturation of another teleost, the red seabream. Since not all the forms of inhibin are available to specifically investigate their autocrine/paracrine role, the apparent conflicting results may be related to both the variations in the form of inhibin present in the preparations, as previously suggested by other researchers, and the species' specific responsiveness to the various forms of gonadal peptides. Thus, taking together all of these findings, it appears that recombinant human inhibin A may have a stimulatory effect in terms of oocyte maturation in some species, while the inhibin used in the other studies, including this one, may have contained a mixture of both inhibin A and inhibin B. Then, we can speculate that inhibin B may be responsible for the inhibition observed on oocyte maturation.

Regarding the activin effects, our findings are consistent with those reported for zebrafish [30] in that recombinant human activin A has a stimulatory effect on *F. heteroclitus *oocyte maturation. However, in contrast to the zebrafish, recombinant human activin A alone did not induce oocyte maturation (GVBD) in *F. heteroclitus*. Nevertheless, recombinant human activin A significantly enhanced DHP-induced GVBD in a dose-dependent manner after 48 hr (Fig. 5). No activin effect was observed during the first 24 hr of culture indicating that activin does not affect the time course of oocyte maturation induced by the steroid, but rather it increases the number of the oocytes undergoing GVBD. In effect, the highest dose of activin used (250 ng/ml) in combination with a low dose of DHP (0.001 μg/ml) raised the GVBD values to a level similar to those induced by a dose of steroid alone ten times higher (0.01 μg/ml). These results support the hypothesis proposed by Pang and Ge [44] that activin may act directly on the oocyte to enhance the maturational competence or responsiveness to DHP in zebrafish. Similarly, it was proposed that activin enhances the oocyte developmental competence in mammals [7]. Activins and other members of the TGFβ initiate their biological actions by interacting with transmembrane receptors named receptor serine kinases (RSKs) [45]. The presence of type I, type II, and type IIB receptors has been demonstrated in a variety of tissues including the ovary, testis, and brain in goldfish and zebrafish [16], thus giving support for the actions of inhibin and activin molecules in teleosts.

*F. heteroclitus*, have group-synchronous ovaries, in which all sizes of vitellogenic follicles are present at any time, and clutches of follicles from a population of oocytes in late vitellogenic stages, are periodically recruited into maturation [46,47]. In order to maintain the integrity of the follicular sequence in this species, it was postulated that local factors other than gonadotropins or steroids might regulate follicle selection into maturation by modulating the oocyte sensitivity to the MIS [48]. Taken together, the in vitro findings from this study indicate that inhibin and activin, which have opposing effects on modulating oocyte maturation and are expressed in the *F. heteroclitus *ovary, are good candidates to serve as local factors in this respect.

## Conclusion

The inhibitory action of inhibin on the FPE stimulation of oocyte maturation was not caused by a decrease in the production of the steroid DHP (the natural inducer of GVBD). Inhibin appears to act directly on the oocyte to block the spontaneous maturation of denuded oocytes, as well as the DHP-induced maturation of intact follicles and denuded oocytes. The inhibitory effect on oocyte maturation was dependent not only on the dose of inhibin, but also on the dose of the stimulatory hormones. High dose of inducing hormone (FPE or DHP) was able to overcome the inhibin inhibition. Activin, on the other hand, significantly enhances DHP-induced GVBD, but does not induce oocyte maturation on its own. Hence, both inhibin and activin are paracrine regulators in the teleost ovary and have opposing effects in the modulation of oocyte maturation.

## Competing interests

The author(s) declare that they have no competing interests.

## Authors' contributions

TRP participated in the design of the study, performed oocyte culture, carried out the statistical analysis and wrote the manuscript. YWL conceived of the study, and participated in its design and coordination, performed the radioimmunoassay (RIA) and helped to draft the manuscript. GT carried out oocyte culture and RIA. All authors read and approved the final manuscript.
